# Mechanical Stress Effects on 550 °C Hot Corrosion Propagation Rates in Precipitation Hardened Ni-Base Superalloys: CMSX-4, CM247LC DS and IN6203DS

**DOI:** 10.1007/s11085-021-10089-w

**Published:** 2021-12-10

**Authors:** Neil Chapman, Simon Gray, Joy Sumner, John Nicholls

**Affiliations:** 1 Siemens Energy Industrial Turbomachinery Limited, Ruston House, Waterside South, PO Box 1, Lincoln, LN5 7FD UK; 2 Cranfield University, College Road, Cranfield, Wharley End, Bedfordshire, MK43 0AL UK

**Keywords:** High-temperature mechanical properties, Strengthening mechanisms

## Abstract

Combinations of temperature, stress and hot corrosion may cause environmentally-assisted cracking in precipitation-hardened Ni-base superalloys, which is little understood. This research aims to increase current understanding by investigating the effects of mechanical stress on the hot corrosion propagation rate during corrosion-fatigue testing of CMSX-4, CM247LC DS and IN6203DS. The parameters used during the tests included a high R-ratio, high frequency, and a temperature of 550 °C. The results showed CMSX-4 experienced a predictable increase in the hot corrosion rate, CM247LC DS also experienced increased rates, but no obvious trend was apparent; whilst IN6203DS showed no evidence of an increased rate. These different behaviours appear to be a result of an interaction between the mechanical stress and microstructural features, which include gamma-prime volume fractions in both the matrix and eutectic regions, along with the distribution of the eutectic structure. The different behaviours in the hot corrosion propagation rate subsequently affected the respective corrosion fatigue results, with both CMSX-4 and CM247LC DS experiencing fracture but with significantly more scatter involved in the CM247LC DS results. All IN6203DS corrosion-fatigue specimens completed the respective tests without fracture and showed no evidence of cracking. It, therefore, appears that precipitation hardened Ni-base superalloys, which are susceptible to environmentally-assisted cracking, also experience increased hot corrosion propagation rates.

## Introduction

Critical rotor blades of an industrial gas turbine (IGT) experience high temperatures and stresses during routine operation. The rotor blades must therefore be manufactured from materials, such as precipitation-hardened Ni-base superalloys, that have favourable high temperature mechanical and oxidation properties. Improvements in these properties have been attained through development of the superalloys [1], allowing the IGT to operate at higher temperatures [2] and efficiencies resulting in reduced CO_2_ emissions [3].

The investment casting technique is used in the manufacture of the rotor blades [3]. This is followed by a suitable heat treatment which ensures a microstructure consisting of minimal gamma-prime eutectic [1] and a homogeneous distribution of gamma-prime precipitates within a gamma matrix [1, 3–5]. The gamma has a disordered fcc unit cell [4, 5] containing elements such as Cr, Co, Re and Mo [6] whilst the gamma-prime is a Ni_3_Al based phase [1] [3] that has an ordered L1_2_ fcc unit cell [3–5]. Other elements associated with the gamma prime are Ta, Ti and Nb which may substitute with the Al to strengthen this phase [1]. In addition, the unit cell of the gamma-prime generally has a smaller lattice parameter to that of the gamma [7], which creates coherency strains [5]. Between the temperatures of 400 to 650 ˚C, the magnitude of these strains increases [6] due to the different thermal expansion rates of the gamma-prime and gamma [7]. Thus, the strengthening mechanisms of the precipitation-hardened Ni-base superalloys include both solid solution and coherency strengthening.

The amount of alloy strengthening tends to increase with the gamma-prime precipitate volume fraction [5] and development of the chemistries [1, 4] has resulted in gamma-prime precipitate volume fractions of up to 70% [3, 6, 7]. This has been achieved by increasing the proportion of elements within the superalloys that are associated with the gamma-prime [6].

The additions of Al and Cr to the chemistries also provide the superalloys with oxidation resistance. That is, a slow-growing protective scale of either alumina or chromia may form, initially alongside transient oxides of all alloying additions. Once the protective scale has formed a continuous layer though, the transient oxides will stop growing since the protective scale acts as a barrier. This prevents O_2_ reacting any further with the elements responsible for the transient oxides [8]. If damaged by cracks or spallation during service [8] though, the protective scales may self-repair [9] providing enough of the respective element (Al or Cr) remains in the superalloy. This self-repair period is known as steady-state oxidation, and during this stage, the rates of oxidation attack are low [9]. Eventually, though, the respective element in the superalloy may become so depleted that the protective scale will no longer be able to self-repair. The superalloy will then enter a breakaway stage and increased rates of oxidation attack will be experienced [9].

In hot corrosion, the self-repair period of the protective scale is known as incubation and may provide limited protection against attack [8, 9]. That is, when molten deposits such as Na_2_SO_4_ accumulate on the surfaces, the protective scale may be damaged [2] by dissolution [8] and thus shorten the incubation stage as the protective scale is forced to self-repair. The Na_2_SO_4_ deposits are in a molten state at a temperature of around 900 ˚C allowing so-called Type I hot corrosion to occur which, having passed through incubation, enters a propagation stage, and causes accelerated internal damage and sulphidation [2, 9, 10]. At temperatures around 700 °C, the Na_2_SO_4_ deposits are in a solid-state but, providing SO_3_ is present in the gas phase, may interact with a transient NiO on the surface of the superalloy and produce a molten Na_2_SO_4_:NiSO_4_ system [2]. This is known as Type II hot corrosion and, during the propagation stage, causes accelerated attack of the superalloy which is characterised by pitting [2, 9, 10].

Hot corrosion may also occur with the deposits remaining in a solid-state [11–13]. An example of this was provided by Kistler et al. [14] After performing hot corrosion exposures on a Ni-base superalloy and repeated on 99.98% pure Ni. These exposures were conducted over various durations (up to 20 h) and used Na_2_SO_4_ deposits (with a surface loading of 2.5 mg cm^−2^) in a gaseous environment of SO_2_ in O_2_ at a temperature of 550 °C. At this temperature, the deposits are not expected to melt when applied to the pure Ni since the Na_2_SO_4_:NiSO_4_ system has the lowest melting point of 671 °C [14]. Similarly, for the superalloy, a Na_2_SO_4_:CoSO_4_ system may form which has the lowest melting point of 565 °C [14] suggesting that this sulphate system is also in the solid-state at 550 °C. For both materials though, accelerated attack did occur. This was attributed to Ni diffusing through NiO and reacting with the deposits to form a metastable nanocrystalline mixed oxide of Na_2_Ni_2_SO_5_, the structure of which allowed rapid Ni^2+^ fluxing.

Similar hot corrosion exposures to those performed by Kistler et al. [14] have also been performed on CMSX-4, CM247LC DS and IN6203DS using a 4/1 molar ratio of Na_2_SO_4_:K_2_SO_4_ deposits, with a surface loading of 0.5 mg cm^−2^ applied every 100 h, in a gaseous environment of 300 ppm SO_2_ in air at 550 °C [15]. Those exposures indicated that a continuous layer of protective scale had failed to establish itself at the relatively low temperature. Despite this, each material still exhibited an incubation and propagation stage. Under these circumstances, the incubation stage may be associated with both the time for the mixed oxide to form and the short-circuit diffusion paths associated with the interfaces of surface-connected refractory metal carbides. Surface roughness analysis indicated the incubation stage took approximately 400, 500 and 200 h for the CMSX-4, CM247LC DS and IN6203DS materials, respectively.

A temperature of 550 °C may therefore allow solid-state diffusion hot corrosion to occur and cause high stresses, which are associated with the increased coherency strains, in the precipitation hardened Ni-base superalloys. These conditions may interact and cause environmentally-assisted cracking (EAC) issues, such as corrosion-fatigue (CF) or stress corrosion cracking (SCC) [16–18], when the mechanical stress associated with the CF or SCC tests is applied. This was investigated by performing a series of stress corrosion exposures on CMSX-4, CM247LC DS and IN6203DS over the temperature range of 450 to 550 °C using the same deposits (4/1 molar ratio of Na_2_SO_4_:K_2_SO_4_ deposits), surface loading (0.5 mg cm^−2^ applied every 100 h) and gaseous environment (300 ppm SO_2_ in air) as stated above. The results indicated a correlation exists between the severity of SCC experienced and the gamma-prime precipitate volume fraction [19]. This led to a new crack initiation/propagation mechanism being proposed that was based on a summation of stresses (which included: applied mechanical stress, surface stress raisers, coherency stresses and stresses associated with interstitial S atoms [20] distorting the lattice structure of the gamma-prime phase), accelerating the hot corrosion attack in a direction that was normal to the applied mechanical stress.

The aim of this research was to provide evidence in support of the proposed new crack initiation/propagation mechanism. That is, does the application of mechanical stress affect the 550 ˚C hot corrosion propagation rate on CMSX-4, CM247LC DS and IN6203DS or not? This was investigated by comparing the respective hot corrosion propagation rates in specimens without the application of mechanical stress [15] against those that were subjected to CF testing using high frequency and high R-ratio parameters.

## Experimental Procedures

### Materials and Test Specimens

Table 1 shows the measured chemistries of the precipitation-hardened CMSX-4, CM247LC DS and IN6203DS Ni-base superalloys that were supplied in the form of bars (5/8″ diameters by 9″ long) that had been cast in the < 001 > orientation. The values shown were obtained from the respective material certificates that generally quoted X-ray florescence results (with the exception of C—which was quoted from LECO analysis results, and Ni—which was stated on the certificates as ‘Balance’ and so has subsequently been arithmetically calculated).

**Table 1:** Superalloys chemistries (wt%) obtained from material certificates

Superalloy	Ni	C	Cr	Co	W	Nb	Ta	Hf	Ti	Al	Re	Zr	Mo
CMSX-4	61.2	0.004	6.5	9.5	6.4	-	6.4	0.1	1.0	5.5	2.8	–	0.6
CM247LC DS	61.5	0.08	8.1	9.3	9.5	-	3.2	1.5	0.7	5.6	–	0.01	0.5
IN6203DS	48.8	0.16	21.4	18.7	2.0	0.8	1.1	1.1	3.5	2.3	–	0.06	< 0.1

The heat treatments of the different materials were performed in accordance with the respective company standards of the sponsor (Siemens Energy Industrial Turbomachinery Limited) of this research. The details of the heat treatments are not included in this paper to protect the sponsors’ heat treatment methodology.

**Fig. 1:**
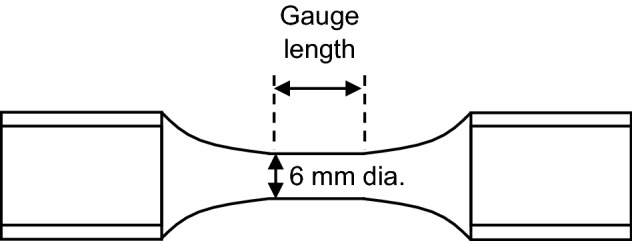
Schematic of fatigue specimen

The materials were single-point turned into plain fatigue specimens (Fig. 1) for the CF tests and 6 mm diameter cylindrical specimens (which represented the gauge diameter of the fatigue specimens) that were 10 mm long for hot corrosion exposures without the application of mechanical stress.

### Hot Corrosion Exposures

Isopropyl alcohol was used to clean the surfaces of the fatigue and cylindrical specimens in an ultrasonic tank. After this, the specimens were thermally exposed to a hot corrosion environment using the deposit recoat practise [9] every 100 h.

The deposits used were a 4/1 molar ratio of Na_2_SO_4_/K_2_SO_4_ that were applied with a surface loading of 0.5 mg cm^−2^ on the outer surface of the cylindrical specimens, and the surface between the threads of the fatigue specimens.

Thermal exposures were carried out at a temperature of 550 °C in a horizontal tube furnace containing an atmosphere of 300 ppm SO_2_ in air that had a flow rate of 50 SCCM. The gas was vented through bubblers using a solution of NaOH as a scrubber.

One cylindrical specimen for each material was exposed for durations of 0, 100, 200, 300, 400, 500, 600, 700 and 800 h. Subsequent surface roughness evaluations [15] indicated each material had entered the propagation stage of hot corrosion after approximately: 400 h for CMSX-4, 500 h for CM247LC DS and 200 h for IN6203DS.

All fatigue specimens were exposed for a total of 500 h before any CF testing commenced. This ensured that each material was plausibly in the propagation stage of hot corrosion (verified by the surface roughness evaluations of the cylindrical specimens) and hence would accelerate the CF tests (since the materials had passed through the hot corrosion incubation stage before the start of the CF tests).

### CF Tests

The CF tests were conducted in load control on a calibrated axial servo-hydraulic load frame that was fitted with a gas chamber. The pre-corroded fatigue specimens were loaded within a pull-bar assembly (within the gas chamber) of the load frame with a gas sheath around the specimen. This gas sheath was heated by an induction coil and thus radiated heat to the fatigue specimen. It also ensured a pre-heated corrosive gas flowed over the surfaces of the fatigue specimen. A type K thermocouple was attached to the fatigue specimen to confirm the required test temperature of 550 °C was achieved.

The corrosive gas used was 300 ppm SO_2_ in air that was pre-heated to the test temperature of 550 °C and flowed at a rate of 25 SCCM. The gas was subsequently scrubbed using a solution of NaOH through bubblers.

The fatigue parameters used included a high R-ratio (*R* = 12/13 which gave high mean stress and thus required relatively small stress amplitudes to prevent the maximum stress exceeding the ultimate tensile strength) and a high frequency. The value of the frequency though is not given in this paper to protect the cycles to failure data for the sponsor of the research (Siemens Energy Industrial Turbomachinery Limited). However, the duration (h) is given for each CF test.

If the fatigue specimen did not fail within 100 h of CF testing, the test was halted and once cooled, the specimen was removed from the load frame. The fatigue specimen would then have a further application of the Na_2_SO_4_/K_2_SO_4_ deposits (0.114 mg cm ^−2^) sprayed evenly on the surface between the threads, before being re-loaded into the load frame for further CF testing. If a fatigue specimen had not failed within three periods of 100 h of testing, the specimen was considered to have achieved a runout and no further CF testing was performed on that specimen.

### Microscopy Techniques

A preliminary visual examination of the CF fracture surfaces was carried out using a Keyence VHX6000 optical microscope. This was followed by a more detailed visual examination using a Jeol JSM-6460 scanning electron microscope (SEM). On completion of the visual examinations, longitudinal and cross-sections (remote from the CF fracture surfaces) of the gauge length were taken, polished to a one-micron finish, and examined on the same SEM in the unetched and electrolytically etched state. The etchant used was a solution of 40 mls of glycerol, 20 mls of hydrofluoric acid and 340 mls of water.

The longitudinal sections that contained cracks remote from the fracture were used to characterise the hot corrosion products within the cracks. This was achieved using Inca software to perform energy dispersive X-ray (EDX) mapping. Owing to overlapping Mo and S X-ray energy peaks though, this technique is unable to differentiate the two elements. However, due to the S content within the sprayed Na_2_SO_4_/K_2_SO_4_ deposits and the relatively small Mo content in the chemistries of the materials, any Mo/S indication was assumed to be S.

Metallographic cross-sections were prepared for the determination of the hot corrosion propagation rates since a true depth of attack could be measured (as opposed to the longitudinal sections where a geometric effect may obscure the true depth). Eight back-scattered images (separated by an angle of approximately 45°) from an etched cross-section of each specimen were obtained. These images were subsequently used to estimate the maximum depth of hot corrosion attack that each fatigue specimen had experienced, which allowed the hot corrosion propagation rate to be calculated as detailed within the Data analysis section.

Secondary electron images were taken from three random areas of polished and etched cross-sections of two fatigue specimens from each material. These images were used to perform image analysis, using Olympus stream motion software, to evaluate the gamma-prime volume fraction associated with the eutectic. The size and volume fraction of the gamma-prime precipitates had previously [19] been performed and are repeated in Table 2 for reference purposes.

**Table 2:** Gamma prime precipitate size and volume fraction data repeated from [19]

Superalloy	Ranking according to severity of cracking	Size (mean ferret, µm)	Volume fraction (%)
CMSX-4	1	0.39	60
CM247LC DS	2	0.46	52
IN6203DS	3	0.12	27

All SEM work was conducted using an accelerating voltage of 20 kV.

### Data Analysis

Cylindrical specimens were used to evaluate the evolution of surface roughness during exposure to the hot corrosion conditions [15]. The data obtained were gathered during a metrology exercise on the polished cross-sections and enabled equations to be derived which provided an estimation of the maximum penetration rate of hot corrosion during the propagation stage of attack. The following were the resulting equations from the data:

1 $$y = 0.0105t - 0.1748$$

2 $$y = 0.00935t + 0.3757$$

3 $$y = 0.0142t + 1.5625$$

where $y$ is the depth of attack (µm) and $t$ is the exposure time to hot corrosion (h).

Equation 1 relates to CMSX-4 material and is applicable between 400 and 800 h (the hot corrosion propagation period under the exposure conditions tested). Similarly, Eq. 2 relates to CM247LC DS material over the period 500 to 800 h and Eq. 3 relates to IN6203DS material which is valid between 200 and 800 h.

Equations 1–3 were therefore used to predict the maximum depth of hot corrosion attack the fatigue specimens had experienced during the 500 h of hot corrosion exposure. This represented the estimated maximum depth of attack at the start of the CF tests.

The eight back-scattered SEM images per fatigue specimen were used to measure the maximum observable depth of hot corrosion attack each fatigue specimen had experienced at the end of the respective CF test. This allowed calculations to be made (based on the equation of a straight line, predicted depth of hot corrosion attack at the start of the CF test, and the duration of the CF test) which estimated the rate of hot corrosion during each CF test. These were subsequently compared with the respective rate component in Eqs. 1–3 to determine if the application of mechanical stress had increased the hot corrosion propagation rate of attack.

## Results and Discussion

**Fig. 2:**
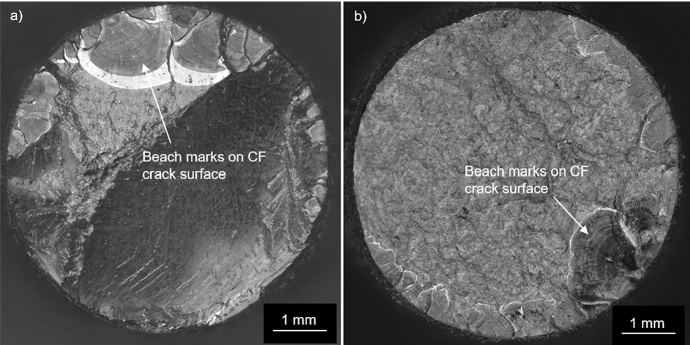
Optical microscopy images showing beach marks observed on the exposed CF crack faces after fracture occurred in **a** CMSX-4 (CF test completed 73 h with mean stress of 820 MPa) and **b** CM247LC DS (CF test completed 99 h with mean stress of 675 MPa)

**Fig. 3:**
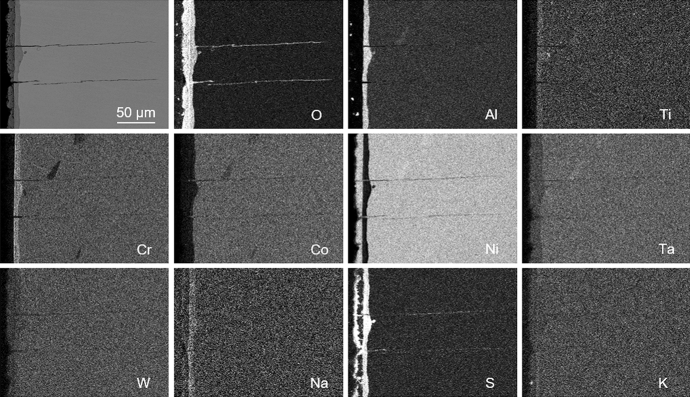
Example of EDX map from an unetched longitudinal section showing O and S penetration into CMSX-4 (CF test completed 73 h with mean stress of 820 MPa)

**Fig. 4:**
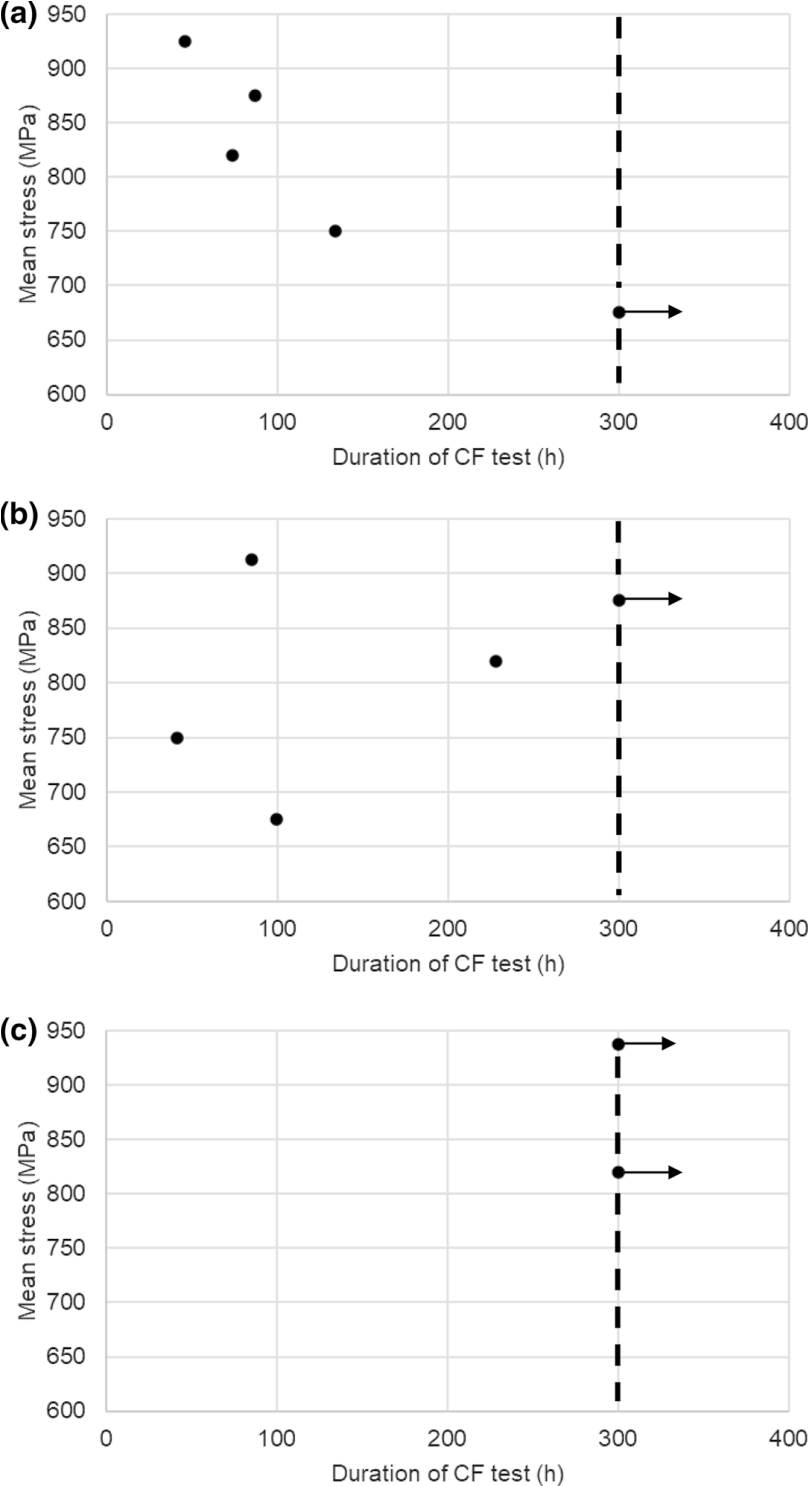
CF results of **a** CMSX-4, **b** CM247LC DS and **c** IN6203DS. Dashed lines represent end of test, arrows indicate runout specimens

Of the three materials subjected to the CF testing, only CMSX-4 and CM247LC DS experienced fractures. The respective fracture surfaces of these two materials indicated similar features in that multiple cracking had occurred which had propagated normal to the direction of mechanical stressing on the < 100 > planes. The exposed crack surfaces revealed beach marks (Fig. 2) suggesting the cracking followed a start-stop-start process, but no evidence of fatigue striations could be found during the SEM examination. EDX mapping, on the unetched longitudinal sections, of the hot corrosion products within the CMSX-4 and CM247LC DS cracks that were remote from the final fracture, revealed an O [21] and S [20] embrittled phase (Fig. 3 shows an example from CMSX-4). Despite these similarities, a comparison of scatter plots showing the effect of mean stress against the duration of testing (Fig. 4) indicated that all three materials displayed different CF behaviours. All the CMSX-4 and CM247LC DS specimens had experienced cracking, which ultimately caused fractures in all but one of these superalloy specimens with significantly more scatter in the CM247LC DS results than in the CMSX-4 results. By contrast, the IN6203DS specimens successfully completed the 300 h of CF testing without showing any evidence of cracking. (For each material, the range of mechanical stress levels that were applied during the respective CF tests, included those which represented elastic stressing and others which represented plastic stressing. The identification of which CF test was performed with elastic or plastic stress though is not given in this paper to protect the sponsors’ design data).

**Fig. 5:**
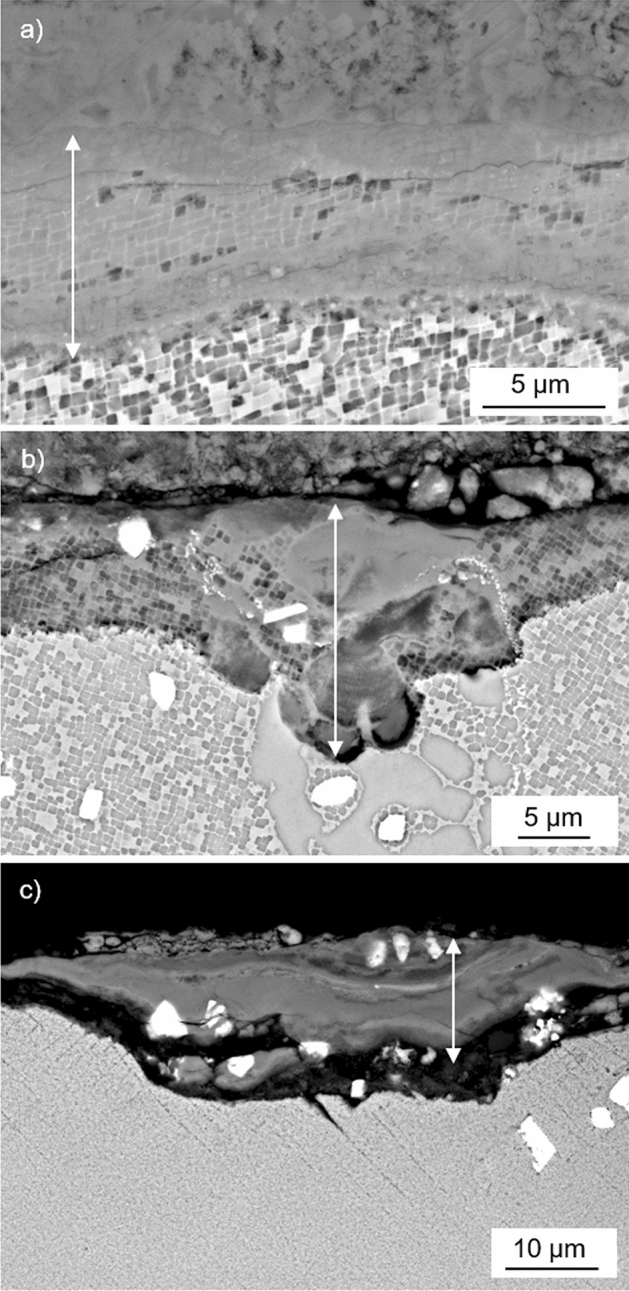
Back-scattered images of adherent intact scale on etched cross-sections of **a** CMSX-4 (CF test completed 86 h with mean stress of 875 MPa), **b** CM247LC DS (CF test completed 40 h with mean stress of 750 MPa) and **c** IN6203DS (CF test completed 300 h with mean stress of 938 MPa). Arrows indicate internal scale as defined by microstructural features (gamma prime and/or carbides)

To understand the different CF behaviours of the three materials, the polished and etched cross-sections of the specimens were used to investigate the effect of mechanical stress on the hot corrosion propagation rate. The back-scattered images obtained indicated the scale present consisted of an external and internal component (Fig. 5) which were defined by the absence or presence of microstructural features such as the gamma-prime precipitates and/or carbides. Further to this, the images indicated the scale to be in various states of damage on each material, which was most likely caused by the high frequency of the CF tests. The damage observed included breakage/cracking of the internal scale in some areas suggesting spallation may have occurred, whilst other areas showed the internal scale to have minimal damage and were largely intact (as indicated in Fig. 5). The maximum observed depth of hot corrosion each fatigue specimen had experienced was therefore based on measuring the thickest intact internal scale from the respective back-scattered images. This allowed the mechanical stress influenced hot corrosion propagation rates, experienced during the CF testing, to be calculated as described in the Data analysis section.

**Fig. 6:**
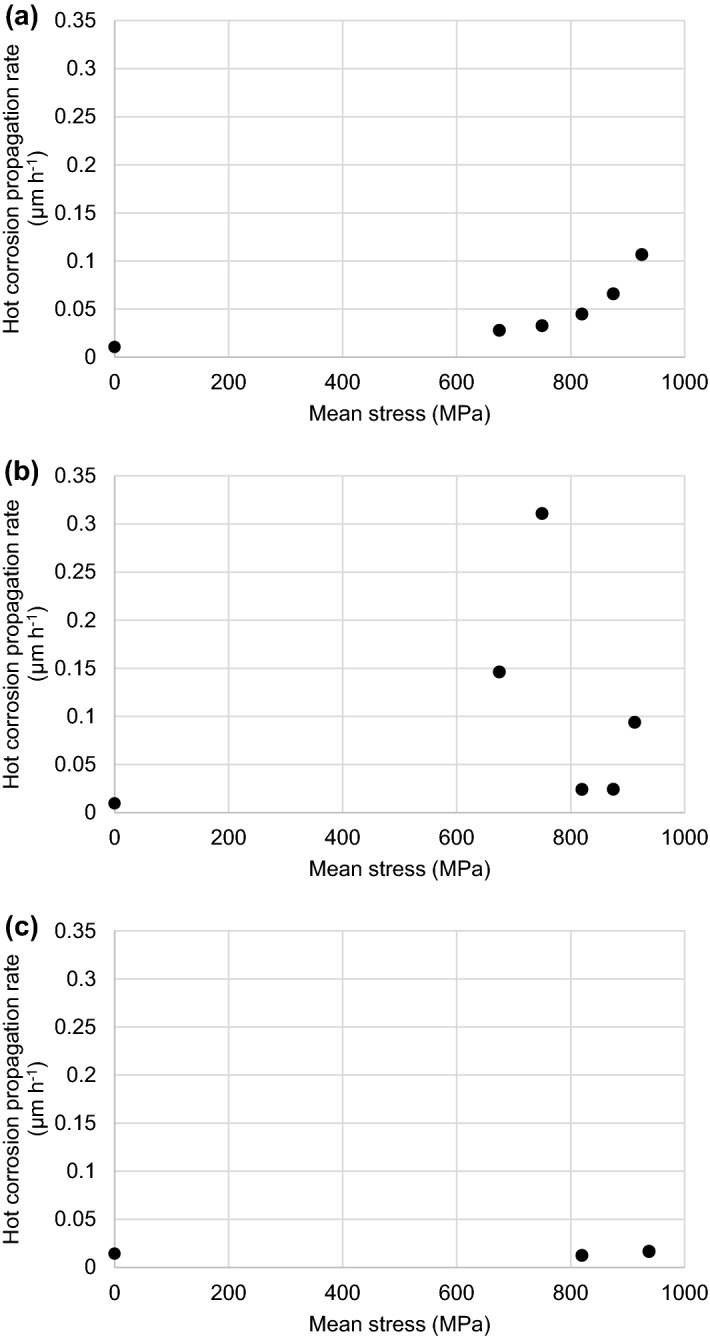
Mechanical stress effect on the maximum hot corrosion propagation rates of **a** CMSX-4, **b** CM247LC DS and **c** IN6203DS

Figure 6 shows a scatter plot for each material illustrating the effect that mechanical stress has on the calculated hot corrosion propagation rates. Also included in these plots is the respective hot corrosion propagation rate from the cylindrical specimens (obtained from the rate component in Eqs. 1–3) which were exposed without any mechanical stress being applied. No significant evidence of an increased hot corrosion propagation rate was found due to the mechanical stress applied during the CF testing of the IN6203DS material (rates of 0.012 and 0.016 µm h^−1^ were calculated which were plausibly equal to the rate component of 0.0142 (µm h^−1^) in Eq. 3). All fatigue specimens manufactured from CMSX-4 and CM247LC DS materials though did experience an increase in the hot corrosion propagation rate during the CF testing when compared with the respective rate components in Eqs. 1 and 2. In the case of CMSX-4, a predictable increasing trend in the calculated rate (rising from 0.028 to 0.107 µm h^−1^) was associated with the mechanical stress which compared with the rate component of 0.0105 (µm h^−1^) in Eq. 1. For CM247LC DS, the rates associated with the CF tests ranged between 0.024 to 0.311 µm h^−1^ which were all greater than the rate of 0.000935 (µm h^−1^) quoted in Eq. 2. However, no obvious trend was apparent for the mechanical stress-influenced CF hot corrosion propagation rates for CM247LC DS, and this appeared to be the cause of the greater degree of scatter in the CF results of this material.

**Fig. 7:**
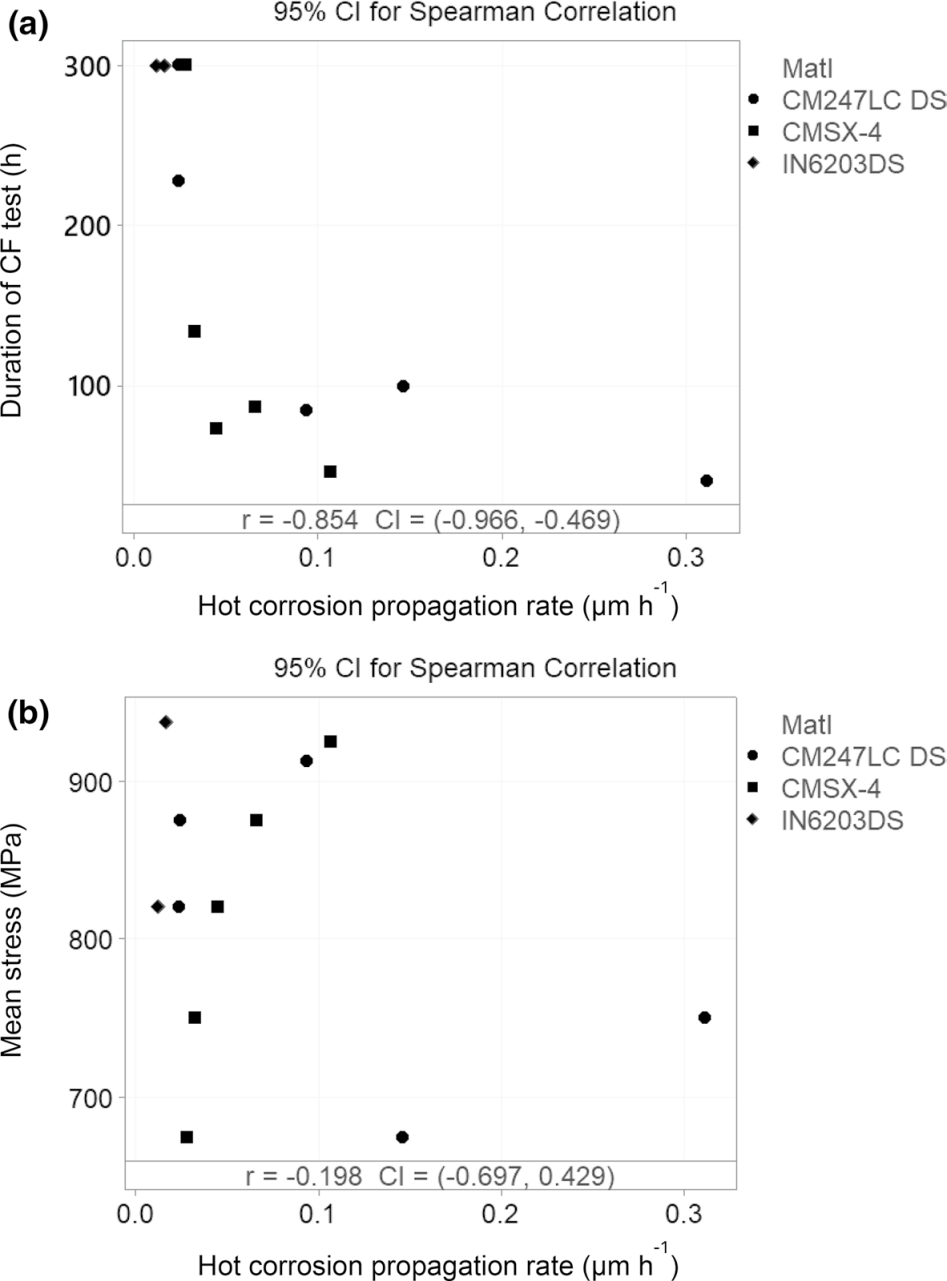
Spearman correlation analysis, performed with a 95% confidence interval, between **a** the hot corrosion propagation rate and the duration of the CF tests, and **b** the hot corrosion propagation rate and the mean stress the CF tests were performed at

After grouping the CF calculated hot corrosion propagation rates of all three materials into one group, a Spearman correlation test [22] was performed (using Minitab version 19 software) against the duration of the CF tests (Fig. 7a). This form of correlation test assesses monotonic relationships between two variables. An output is then provided which is used to determine if a non-linear relationship plausibly exists between the two variables. The correlation coefficient obtained (− 0.854 with a 95% confidence interval of − 0.966 to − 0.469) indicated a strong relationship suggesting the hot corrosion propagation rate is an influential factor in the CF life of the three materials tested. However, it does not explain the cause of the increased rates.

Another Spearman correlation test was performed (Fig. 7b), but this time against the CF mean mechanical stress and the CF hot corrosion propagation rates. The correlation coefficient obtained (− 0.198 with a 95% confidence interval of − 0.697 to + 0.429) indicated little evidence for a relationship between these two variables. Although this may suggest that mechanical stress may not be an influential factor in the CF tests, it does not preclude it from interacting with another factor such as the microstructure.

Table 2 indicates the gamma-prime precipitate volume fraction is correlated with the ranked severity of cracking in SCC tests. The gamma-prime, therefore, appears to be a susceptible phase to EAC and this suggests the gamma-prime associated with the precipitates and/or eutectic features may be interacting with the mechanical stress.

The exact mechanism involved in the interaction is presently unknown. However, one possibility may concern the size of the gamma-prime interstitial sites and the relative size of interstitial species such as S. In the unstressed state, it may be that the interstitial sites are too small for S to gain access. In a mechanically stressed state though, the interstitial sites may have been distorted to such an extent that S can access these sites. This would then further distort the gamma-prime lattice and increase the summation of stresses associated with the gamma-prime. The gamma lattice would also experience distortion under the application of mechanical stress. However, since the gamma tends to have a larger lattice parameter than that of the gamma-prime, the effect of the interstitial S further distorting this lattice structure may not be as great as that for the gamma-prime. This would therefore ensure that the gamma-prime is the susceptible phase. Significant research is required though, before any evidence (for or against) this possibility is obtained.

The interaction between the three factors (mechanical stress, gamma-prime precipitate volume fraction and gamma-prime eutectic volume fraction) may be more influential than the individual factors alone. Evidence of this possible interaction can be seen in Fig. 5b, which shows a localised region, associated with the gamma-prime of a surface connected eutectic region, that has experienced greater depths of hot corrosion attack than the adjacent areas.

**Fig. 8:**
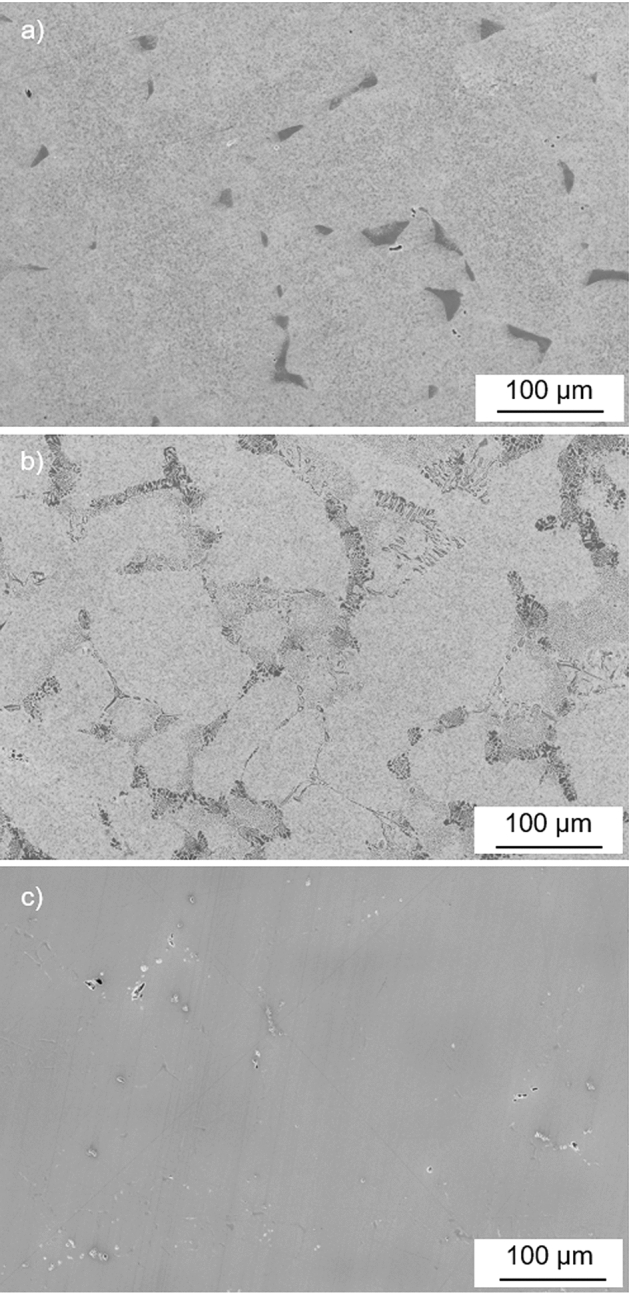
Secondary electron images of etched cross-sections showing the gamma prime eutectic in (**a**) CMSX-4 with a volume fraction, in image shown, of 3.0% (CF test completed 300 h with a mean stress of 675 MPa), **b** CM247LC DS with a volume fraction, in image shown, of 8.0% (CF test completed 300 h with a mean stress of 875 MPa) and **c** IN6203DS with a volume fraction, in image shown, of 0% (CF test completed 300 h with a mean stress of 820 MPa)

To determine the gamma-prime eutectic volume fraction of the CMSX-4, CM247LC DS and IN6203DS materials, image analysis was performed (using Olympus Stream motion software) on three random areas of the etched cross-sections of two CF tested specimens per material. Table 3 shows the respective volume fraction for each area. For IN6203DS, no evidence of gamma-prime eutectic could be found in either specimen. In the case of CMSX-4, one specimen had gamma-prime eutectic volume fractions of 1.2, 0.9 and 1.5% (averaging out at a mean value of 1.2%) whilst the other specimen had values of 3.0, 2.1 and 3.3% (mean average of 2.8%). For CM247LC DS, values of 8.0, 6.3 and 6.1% (mean average of 6.8%) were recorded in one specimen and 7.7, 5.9 and 6.8% (mean average of 6.8%) were recorded in the other specimen. The overall mean average of the gamma-prime volume fraction within the eutectic features of the two specimens per material that contained the eutectic features was 2.0% for CMSX-4 and 6.8% for CM247LC DS. This indicates that the gamma-prime eutectic volume fraction was more than three times greater in CM247LC DS than that in CMSX-4. The distribution of the gamma-prime eutectic also differed between the two materials. In the case of CMSX-4 the gamma-prime eutectic appeared as isolated ‘clumps’ within the microstructure whilst that in CM247LC DS tended to be strung out around the dendritic features (Fig. 8). Providing the gamma-prime eutectic was surface connected, the relative distributions would allow the hot corrosion a potentially easier path to track and thus result in greater localised propagation rates for CM247LC DS than that for CMSX-4 during the CF tests. Hence, the distribution of the gamma-prime eutectic maybe a fourth factor which is involved in the interaction which causes the increased hot corrosion propagation rates during the CF testing (along with mechanical stress, gamma-prime precipitate volume fraction and gamma-prime eutectic volume fraction).

**Table 3:** Gamma prime eutectic volume fraction (%) found within the CF specimens

Superalloy	Mean stress of CF test (MPa)	Random area 1	Random area 2	Random area 3	Mean average
CMSX-4	875	1.2	0.9	1.5	1.2
CMSX-4	675	3.0	2.1	3.3	2.8
CM247LC DS	875	8.0	6.3	6.1	6.8
CM247LC DS	750	7.7	5.9	6.8	6.8
IN6203DS	820	0	0	0	0
IN6203DS	938	0	0	0	0

Of the four factors which are proposed in the influential interaction that causes an increase in the hot corrosion propagation rates, and ultimately CF failures, the dominant factor(s) generally appear to be the ones associated with the microstructure. In the case of IN6203DS, the relatively low gamma-prime content (volume fractions of 27 and 0% for the precipitate and eutectic respectively) meant this material did not appear to experience any increase in the hot corrosion propagation rates with concurrent fatigue testing and was therefore immune to the influence of mechanical stress (under these conditions). CMSX-4 had gamma-prime volume fractions of 60 and 2.0% for the precipitate and eutectic (which appeared as isolated ‘clumps’) respectively. It seems that the eutectic volume fraction and distribution were insufficient to exert any major influence on the interaction allowing the magnitude of the mechanical stress to become the dominant factor in the predictable increase in the hot corrosion propagation rates as shown in Fig. 6. CM247LC DS had gamma-prime volume fractions of 52 and 6.8% respectively for the precipitate and eutectic (which tended to be a continuous distribution around the dendritic structure). In this case, it appears that the magnitude of stressing became less dominant and the eutectic volume fraction and distribution more influential within the interaction. This introduced a greater degree of randomness within the maximum hot corrosion propagation rates (Fig. 6), which was most likely due to how many and how deep the surface-connected gamma-prime eutectic features were within the gauge length of the fatigue specimens. This randomness was therefore the most likely cause of the scatter observed within the CF results of CM247LC DS material (Fig. 4).

The results of this research have provided evidence that the application of mechanical stress may increase the hot corrosion propagation rates in precipitation-hardened Ni-base superalloys. However, it does appear to be dependent on an interaction with microstructural features which include the gamma-prime precipitate and eutectic volume fractions and the distribution of the eutectic features. Once increased rates of the hot corrosion propagation rate have been experienced, the respective material may be considered more likely to experience EAC issues such as CF (using high R-ratio and high-frequency parameters) or SCC.

## Conclusions

Upon completion of this research, the following conclusions were made:

- Precipitation hardened Ni-base superalloys that appear susceptible to EAC are those with relatively high gamma-prime (precipitate and/or eutectic) volume fractions.
- IN6203DS (with averaged gamma-prime volume fractions of 27 and 0% for the precipitate and eutectic respectively) does not appear to be susceptible to EAC.
- CMSX-4 (with averaged gamma-prime volume fractions of 60 and 2.0% for the precipitate and eutectic respectively) and CM247LC DS (with averaged gamma-prime volume fractions of 52 and 6.8% for the precipitate and eutectic respectively) are both susceptible to EAC.
- EAC susceptible precipitation hardened Ni-base superalloys appear to experience an increased hot corrosion propagation rate due to an interaction between mechanical stress, the gamma-prime precipitate and eutectic volume fractions, and the distribution of the gamma-prime eutectic.
- One possible explanation for the interaction is that the mechanical stress may be distorting the lattice structure of the gamma-prime sufficiently for S to access the interstitial sites within these phases. This would then create a further distortion of the gamma-prime lattice and therefore allow the S to access more interstitial sites. Further to this, if relatively large eutectic regions of the gamma-prime are surface connected, the increased hot corrosion rates would ensure localised deep penetration of the hot corrosion attack in a relatively short time frame. The lattice structure of the gamma matrix though would also be distorted by the mechanical stress. However, since the lattice parameter of the gamma-prime tends to be smaller than that of the gamma matrix, any further distortion caused by S accessing the interstitial sites would be of greater magnitude in the gamma-prime ensuring these are the susceptible phases. This is an area that should be considered for further research by computer modelling. It may also be possible that a critical gamma-prime precipitate size for a given volume fraction may be derived below which increased rates of hot corrosion attack due to mechanical stress do not occur.
- This research has shown that a proposed crack initiation/propagation mechanism based on a summation of stresses accelerating the 550 ˚C hot corrosion attack is plausible in susceptible precipitation hardened Ni-base superalloys.

## Declarations

### Availability of Data and Material

The data cannot be shared at this time as it is part of an ongoing study.
